# A Practical Text-Book of the Diseases of Women

**Published:** 1892-09

**Authors:** 


*A Practical Text-book of the Diseases of Women.* By ARTHUR H. N. LEWERS, M.D. Lond. Third Edition. Pp. xx., 454. London: H. K. LEWIS. 1891.

The third edition of this book presents few differences from the second. The chapter on hydatid mole is extended and amplified, and the number of illustrative cases has been considerably increased, rendering the book more interesting and valuable. One or two sketches illustrating the different steps of Tait's operation for the repair of the ruptured perineum might have been inserted with advantage, as it is by no means easy for one, unacquainted with the operation practically, to follow the description by means of the diagram given. The operation, simple enough in itself, is difficult to describe clearly to those who have never seen it performed; but a couple of drawings illustrating the different stages would make it quite clear. We also miss any reference to the use of the Apostoli treatment in cases other than fibroids.

The book is, however, an excellent and concise manual, and will be found especially useful by students preparing for examination.

